# The Phosphoproteomic Response of Rice Seedlings to Cadmium Stress

**DOI:** 10.3390/ijms18102055

**Published:** 2017-09-27

**Authors:** Min Zhong, Sanfeng Li, Fenglin Huang, Jiehua Qiu, Jian Zhang, Zhonghua Sheng, Shaoqing Tang, Xiangjin Wei, Peisong Hu

**Affiliations:** 1 State Key Lab of Rice Biology, China National Rice Research Institute, Hangzhou 311400, China; zhongmin2007@163.com (M.Z.); lsanfeng@126.com (S.L.); 19phoenix82@163.com (F.H.); qiujiehua@caas.cn (J.Q.); zhangjian@caas.cn (J.Z.); shengzhonghua@caas.cn (Z.S.); qstang@126.com (S.T.); 2 College of Agronomy, Jiangxi Agricultural University, Nanchang 330045, China; 3 College of Agronomy, Hunan Agricultural University, Changsha 410128, China

**Keywords:** cadmium, rice (*Oryza sativa* L.), iTRAQ, phosphoproteome, post-translational modification

## Abstract

The environmental damage caused by cadmium (Cd) pollution is of increasing concern in China. While the overall plant response to Cd has been investigated in some depth, the contribution (if any) of protein phosphorylation to the detoxification of Cd and the expression of tolerance is uncertain. Here, the molecular basis of the plant response has been explored in hydroponically raised rice seedlings exposed to 10 μΜ and 100 μΜ Cd^2+^ stress. An analysis of the seedlings’ quantitative phosphoproteome identified 2454 phosphosites, associated with 1244 proteins. A total of 482 of these proteins became differentially phosphorylated as a result of exposure to Cd stress; the number of proteins affected in this way was six times greater in the 100 μΜ Cd^2+^ treatment than in the 10 μΜ treatment. A functional analysis of the differentially phosphorylated proteins implied that a significant number was involved in signaling, in stress tolerance and in the neutralization of reactive oxygen species, while there was also a marked representation of transcription factors.

## 1. Introduction

The heavy metal cadmium (Cd) is phytotoxic; the exposure of roots to relatively low concentrations of Cd^2+^ are sufficient to suppress enzyme activity, inhibit photosynthesis, block stomatal movement and interfere with transpiration, then result in leaf roll, growth inhibition and chlorosis [1,2]. Cd, as a non-essential element of plant, is readily taken up by the root through the action of transporters used by the plant to import various essential micro- and macroelements, thereby reducing the plant’s capacity to take up, transport and utilize these essential elements [3,4]. The presence of Cd^2+^ ions disturbs the plant’s water balance [5], inhibits oxidative mitochondrial phosphorylation [6], interferes with normal H^+^/K^+^ exchange, down-regulates the activity of plasma membrane ATPase [7] and indirectly increases the level of oxidative stress [8]. Plants have evolved a range of protective measures, including the pumping of ions into the apoplast, their extracellular immobilization, their chelation in the cytosol and their sequestration into the vacuole [9,10]. The products of a number of genes have been implicated in these defense and tolerance processes [11,12,13]. More recently, transcriptomic, proteomic and metabolomic platforms have been applied to characterize the Cd response. For example, a comparison in pakchoi (*Brassica chinensis*) between varieties which contrast for their uptake of Cd was able to show that the timing of certain transcriptional responses was variety dependent [14]. In cotton, Cd stress boosts the production of enzymes involved in the neutralization of reactive oxygen species (ROS), in mitochondrial respiration and in lignification [15]. A combined transcriptomic and metabolomic analysis of radish roots exposed to Cd has revealed effects on the plants’ amino acid metabolism, energy production and oxidative phosphorylation [16].

It is well understood that post-translational modification is exploited in order to regulate protein activity. Over 300 types of such modifications are known, the major ones being glycosylation, acetylation, phosphorylation and nitrosylation. The contribution of phosphorylation is the most fully explored of these, thanks to its relatively high stability, abundance and functional importance. Phosphorylation affects most commonly the hydroxyl group in threonine, serine and tyrosine [17]. A large number of signaling pathways involved in the response of rice to Cd stress are known to be mediated by phosphorylation/dephosphorylation events [18,19,20], although the level of understanding of these events is limited by the universal focus on a single phosphoprotein [20,21]. Technological developments in phosphoproteomics now offer novel opportunities to identify phosphosites more globally. Thus, large scale scans of induced phosphoproteins have been carried out with a view to characterizing the plant response to drought [22], exogenous hormone treatment [23,24], salinity [25] and high temperature [26], although not as yet to heavy metal exposure. The current paper presents a comprehensive analysis of phosphorylation induced in rice seedlings by their exposure to Cd, conducted using an iTRAQ (isobaric tag for relative and absolute quantitation) based quantitative phosphoproteomic approach.

## 2. Results

### 2.1. The Phenotypic and Physiological Effects of Exposure to Cd^2+^ Stress

**Figure 1:**
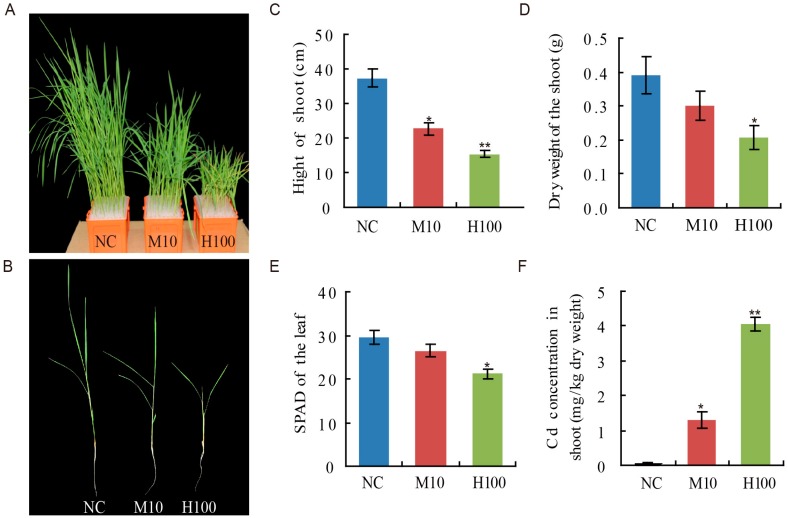
The morphology of the rice seedling shoot and its Cd content when grown hydroponically for 12 days in a solution containing 0 μΜ (NC), 10 (M10) μΜ or 100 μΜ (H100) Cd^2+^. (**A**,**B**) The appearance of the seedlings; (**C**) the effect on shoot height; (**D**) the effect on dry matter accumulation; (**E**) the effect on the leaf chlorophyll content (soil and plant analyzer development, SPAD) after 12 days of Cd^2+^ treatment; (**F**) the effect on tissue Cd content. Values in (**C**–**F**) are means ± SD (*n* = 3); the asterisks indicate statistical significance between the M10, H100 and NC, as determined by a Student’s *t*-test (* *p* ≤ 0.05; ** *p* ≤ 0.01).

Increasing the concentration of Cd^2+^ present in the hydroponics solution had a suppressive effect on the growth of rice seedlings (Figure 1A–E). Compared to the performance of non-stressed (control, NC) seedlings, seedling height after a 12 day exposure to Cd was reduced by 42.9% in the 10 μΜ Cd^2+^ treatment (M10) and by 60.8% in the 100 μΜ Cd^2+^ treatment (H100). Whereas the control seedlings had accumulated 0.39 g of dry matter by the time of harvest, the dry weight of the stressed seedlings was only 0.30 g (M10) and 0.21 g (H100) (Figure 1D). Both Cd treatments significantly decreased the seedlings’ chlorophyll content (Figure 1E). In addition, the shoots’ Cd content of seedlings in both M10 and H100 were also very significantly raised (Figure 1F).

### 2.2. The Identification of Phosphorylated Proteins and Phosphosites

**Figure 2:**
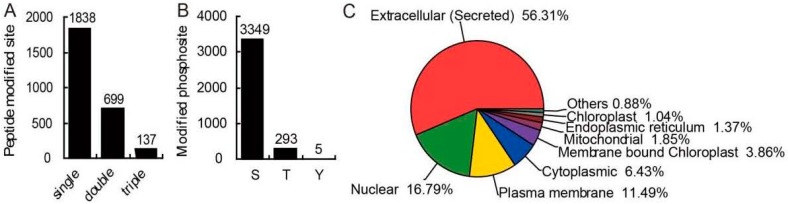
Phosphosite types and the subcellular location of phosphoproteins. (**A**) The frequency of phosphopeptides carrying one, two or three phosphosites; (**B**) The distribution of phosphosites between serine (S), threonine (T) and tyrosine (Y) residues; (**C**) The putative subcellular location of the set of phosphoproteins.

The contribution of protein phosphorylation to the response of rice seedlings to Cd stress was derived by conducting an iTRAQ-based phosphoproteomic analysis. A total of 2681 unique phosphopeptides (Figure 2A; Table S1) was identified, associated with 1244 proteins (Table S2). Some 68.8% of the peptides were modified at a single site, 26.1% were altered at two and the remainder at three sites (Figure 2A). A total of 3647 phosphosites detected, of them, 3349 (91.8%) involved a serine residue, 293 (8.0%) a threonine and just five (0.1%) a tyrosine (Figure 2B). Our research found a similar distribution pattern of the phosphorylation types with other reports in *Triticum aestivum*, rice and *Brachypodium distachyon* [27,28].

### 2.3. Predicted Subcellular Localization of Phosphoproteins

The putative subcellular localization of the phosphoproteins was derived by an in silico analysis based on a subcellular localization prediction online tool (Available online: http://cello.life.nctu.edu.tw/) [29,30]. Extracellular phosphoproteins accounted for >56.31% of the set of phosphoproteins (Figure 2C); Of the remainder, 11.49% were predicted to be associated with the plasma membrane, 16.79% with the nucleus and 6.43% with the cytoplasm, with the rest 9.00% being distributed among the mitochondria, the chloroplasts, the endoplasmic reticulum and the others.

### 2.4. Peptide Motifs Associated with Phosphorylation

**Figure 3:**
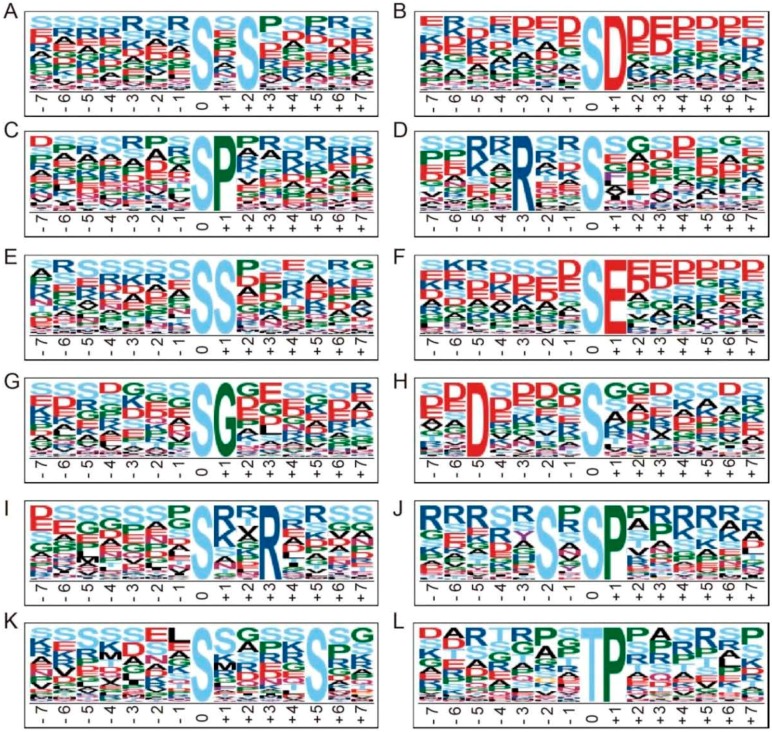
Motif-X analysis of over-represented motifs around the phosphosites. (**A**) ‘sxS’; (**B**) ‘sD’; (**C**) ‘sP’; (**D**) ‘Rxxs’;(**E**) ‘sS’; (**F**) ‘sE’; (**G**) ‘sG’; (**H**) ‘Dxxxxs’; (**I**) ‘sxxR’; (**J**) ‘SxsP’; (**K**) ‘sxxxxS’; (**L**) ‘tP’. In motif names, the phosphoserine or phosphorthreonine residue at the position 0 were represented as “s” or ”t”; the conservative and unconservative residues around the phosphosite were represented as uppercase and “x”, respectively.

A set of 2454 distinct sequences representing the 15 residue surrounding each of the phosphosites was obtained. Of these, 2404 were centered on a serine residue and 50 on a threonine (Table S3, Figure 3). The former set included twelve over-presented motifs: the most common ones were ‘sxS’ (476 occurrences) and ‘sD’ (385 occurrences), followed by ‘sP’ and ‘Rxxs’ (each with >200 occurrences). The ‘sP’ motif has also been identified as being over-represented in other systems [31,32,33]. ‘sP’ is recognized by MAPK (mitogen-activated protein kinase), SnRK2 (sucrose non-fermenting1-related protein kinase 2), AGC (cAMP and cGMP dependent protein kinase C), RLK (receptor-like kinase), CDK (cyclin-dependent kinase), STE20-like kinase (SLK) and CDPK (calcium-dependent protein kinase) kinases [31]. Meanwhile the ‘Rxxs’ motif provides a target for MAPKK (Mitogen-activated protein kinase kinase), protein kinase A and CaMK-II (Calmodulin-dependent protein kinase) [31,33]. There were over 50 occurrences of each of ‘sS’, ‘sE’, ‘sG’, ‘Dxxxxs’, ‘sxxR’, ‘SxsP’, ‘sxxxxS’ and ‘Kxxs’. As for phosphothreonine, ‘tP’ was found to be the only conserved motif in this study.

### 2.5. Differentially Phosphorylated Proteins in Response to Cd^2+^ Treatment

**Figure 4:**
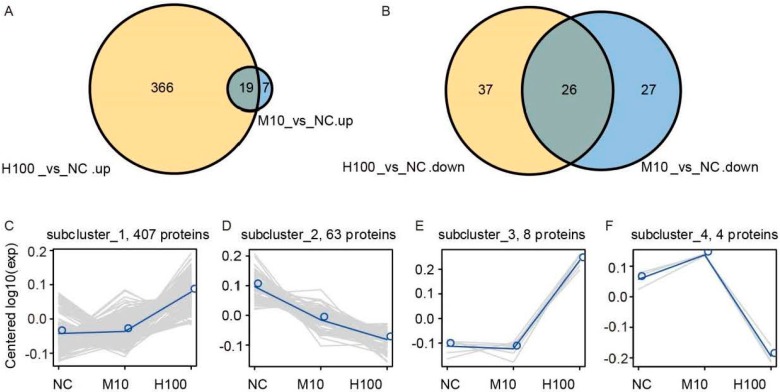
The expression of the 482 identified differentially phosphorylated proteins identified by iTRAQ. (**A**) Phosphorylated proteins up-regulated differentially by the presence of Cd^2+^; (**B**) Phosphorylated proteins down-regulated differentially by the presence of Cd^2+^. The orange segments refer to proteins more abundant in the H100 treatment but not in the M10 treatment, and the blue ones vice versa; the gray segments refer to proteins differentially abundant in both Cd^2+^ treatments; (**C**–**F**) Contrasting patterns of expression among the 482 differentially phosphorylated proteins: within each panel, the gray lines represent the abundance of individual phosphoproteins, and the light blue line represents the average pattern.

Differential phosphorylation was inferred whenever the presence of Cd^2+^ altered the abundance of a phosphorylated protein by at least two fold (*p* ≤ 0.05). Of the 1244 proteins identified in the seedlings, 482 fell into this category: 403 of these were associated with the H100 treatment and 34 with the Μ10 treatment, while 45 featured in both treatments (Figure 4A,B, Tables S4 and S5). Of the 392 proteins up-regulated by the stress, 366 were identified in the H100 treated seedlings, 7 in the M10 treated seedlings and 19 in both treatments (Figure 4A). The H100 treatment suppressed 37 of the differentially phosphorylated proteins, the M10 treatment suppressed 27, and 26 were suppressed in both treatments (Figure 4B). Overall, the abundance of 407 proteins was enhanced in both treatments and that of 63 was reduced; a small number (12) of proteins was up-regulated in one treatment but down-regulated in the other (Figure 4C–F).

### 2.6. Functional Assignment of the Differentially Phosphorylated Proteins

**Figure 5:**
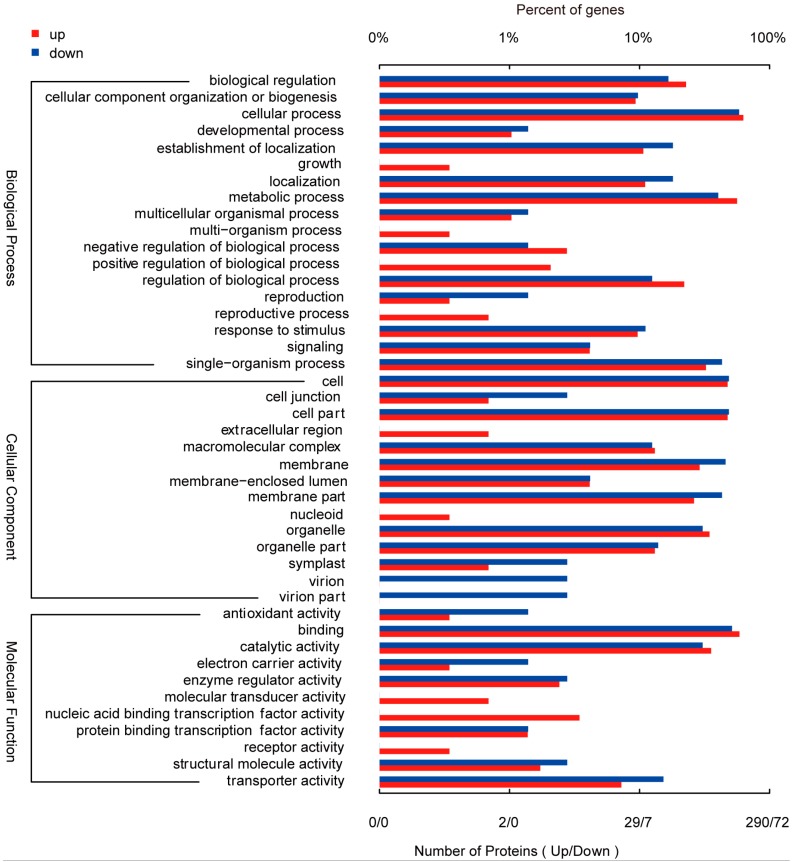
Gene ontology analysis of the set of differentially phosphorylated proteins. The red and blue bars represent proteins which were, respectively, increased and decreased in abundance by exposure to Cd stress.

A set of differentially phosphorylated (DP) proteins was subjected to a gene ontology (GO) analysis (Figure 5). For “biological process”, DP proteins related to cellular process, metabolic process and single-organism process were preferred changed in response to Cd stress, whereas growth and multi-organism process was under-presented. Regarding “cellular component”, DP proteins associated with cell, cell part and membrane part were up-represented, but nucleoid and extracellular region were less presented. Finally, from the “molecular function” perspective, DP proteins involved in binding, catalytic activity and transporter activity were over-presented, while receptor activity and molecular transducer activity were less preferred. When separately conducted GO enrich analysis with DP proteins in M10 or H100, there was significant different in the categories of “biological process”, “cellular component” and “molecular function” (Figures S1 and S2). The phosphoproteins displaying the greatest change in abundance as a result of the M10 treatment fell into the “cellular component” categories “membrane part”, “transcription factor complex” or “nucleolar part”, whereas the genes modulated by H100 fell largely into the categories “vacuole”, “mitochondrial matrix” or “endomembrane system”. In terms of “molecular function”, the M10 treatment induced proteins for the most part involved “binding of cytoskeletal protein”, “microtubule and tubulin”, “oxidoreductase activity” or “transporter activity”, while the H100 treatment induced proteins associated with “sucrose synthase activity”, “binding of histone” or “Rab (Ras-related in brain) GTPase”. Finally, with respect to “biological process”, the majority of the proteins induced by M10 were categorized as a “cellular process”, “metabolic process of carbohydrate”, “homeostatic process” or “response to oxidative stress”, while the H100-induced ones were mostly assigned to “regulation involving in cellular component”, “vesicle fusion” or “transcription”.

### 2.7. The Abundance of Transcript from Genes Encoding Differentially Phosphorylated Proteins

**Figure 6:**
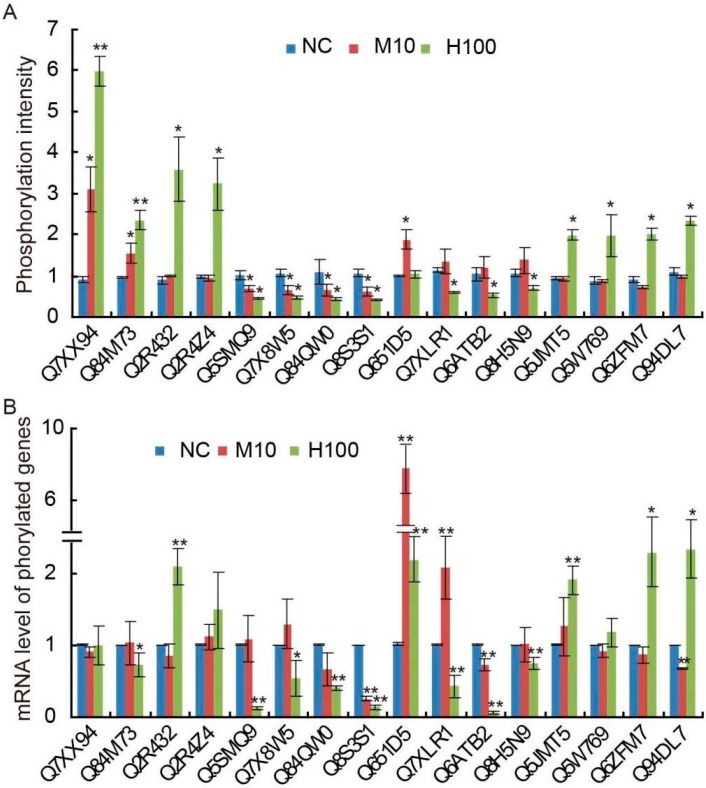
The relationship between (**A**) phosphorylation intensity and (**B**) transcript abundance, based on the response of 16 genes encoding differentially phosphorylated proteins. Values are means ± SD (*n* = 3); the asterisks indicate statistical significance between the M10, H100 and NC, as determined by Student’s *t*-test (* *p* ≤ 0.05; ** *p* ≤ 0.01).

An attempt was made to establish a correspondence between the transcript abundance of randomly selected 16 genes encoding a differentially phosphorylated protein and the degree of their phosphorylation; the former was derived using a quantitative real time PCR (qRT-PCR) assay (Figure 6A,B). The result showed that there was no evidence for any correlation for eleven of the proteins, which indicated that the protein phosphorylation events of rice shoot in response to Cd stress are independent of the protein amount. For example, both Q7XX94 and Q84W73 became highly phosphorylated in response to the H100 treatment, but there was no up-regulation in the transcription of their encoding genes. The proteins Q5SMQ9, Q7X8W5 and Q84QW0 all experienced a loss in phosphorylation in response to the M10 and H100 treatment, but the transcription of these proteins did not change significantly in Μ10. The abundance of transcript produced by the genes encoding Q2R4Z4, Q5W769 and Q651D5 was unrelated to the extent of their phosphorylation in response to the Μ10 treatment, while the abundance of Q8S3S1, Q6ATB2 and Q7XLR1 transcript was reduced more strongly than the extent of their dephosphorylation induced by Cd stress.

### 2.8. Protein-Protein Interactions Involving Differentially Phosphorylated Proteins

**Figure 7:**
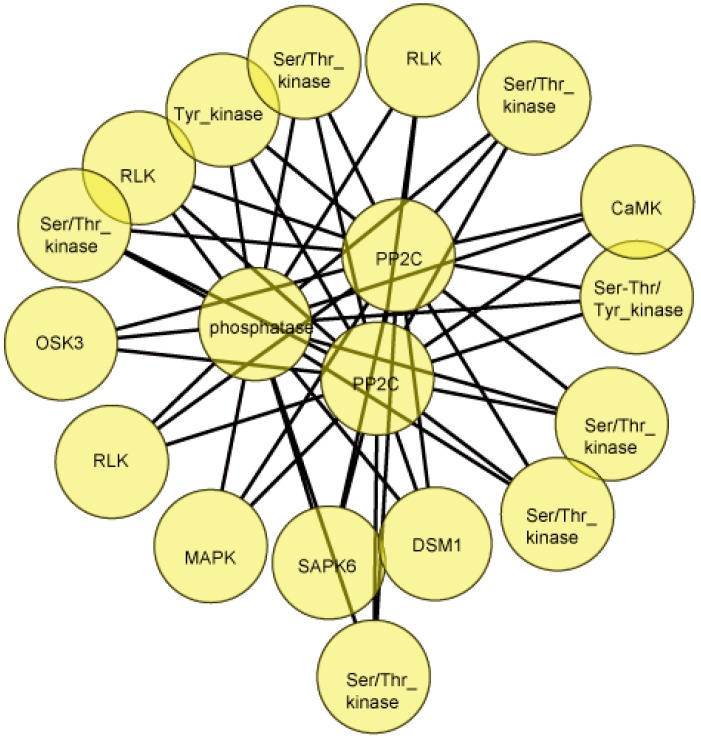
A network analysis of the set of differentially phosphorylated kinases and phosphatases. The full form of the abbreviated ID’s is given in Table S6.

An interaction network based on the set of differentially phosphorylated proteins comprised of 19 nodes and 44 edges (Figure 7, Table S6). An analysis focused on just kinases and phosphatases revealed the centrality of three protein phosphatases belonging to the PP2C (type 2c protein phosphatase) family, which implied that abscisic acid (ABA)-related signaling is likely of some importance for Cd detoxification and tolerance. For example, the self-phosphorylating SAPK6 (stress-activated protein kinase) has been associated with both the response to osmotic stress and ABA signaling [34]. Additionally, phosphorylated proteins involved in MAPK (mitogen-activated protein kinase) and CaMK (calmodulin dependent protein kinase) systems were also included in the interaction network. For instance, DSM1 (drought-hypersensitive mutant1) is thought to be a MAPK kinase kinase functioning as an early signaling component the drought stress response [35]. The presence of Q10BA4/CaMK in the network suggested that CaMK also participate in the response to Cd stress.

## 3. Discussion

### 3.1. Protein Kinases/Phosphatases Participated in Signal Perception and Transduction

The ABA signaling pathway is of major importance to the plant response and defense to Cd stress. It was revealed that ABA was rapidly induced by Cd^2+^ in *S. tuberosum* [36]. Exogenous ABA was reported to not only decrease Cd content in *O. sativa* by ABA-assisted reduced Cd root-to-shoot translocation via decreased transpiration rate [37], and confer Cd tolerance as result of increase of phytochelatins content through increased *O*-acetylserine (thiol) lyase and phytochelatin synthase [38,39]. The central signaling complex of ABA pathway has been shown to be formed by the interaction of pyrabactin resistant protein (PYR), PYR-Like protein (PYL), Regulatory components of ABA receptors (RCAR), PP2Cs and SnRK2s; PP2Cs relieve the inhibition of SnRK imposed by their phosphorylation by competitively binding with ABA receptors [40]. Here, the phosphorylation level of two of the three known PP2Cs (PP2C66 and PP2C30) and three SnRK1s (Q6ZI44/Os02g0551100, Q0J0U2/Os09g0499000 and Q18PR9/Os03g0319100) was increased in response to the H100 treatment (Tables S4 and S5). The phosphorylation of PP2C66 and PP2C30 likely promotes their binding to ABA receptors, enhancing the level of phosphorylation of Q6ZI44, Q0J0U2 and Q18PR9, which in turn promotes the phosphorylation of AREB/ABF (ABA-responsive element-binding protein/ABA-responsive element-binding factors) and the induction of genes related to Cd translocation and tolerance; This hypothetical model is consistent with the behavior both of wheat plants exposed to drought [22] and of rice plants to infection by bacterial blight [27]. Two ARFs (Q94DL7/Os01g0963600 involved in aluminum tolerance [41,42] and Q2R4Z4/Os11g0454300 in salinity tolerance [43]) were identified here as experiencing enhanced phosphorylation involving both the Ser41/73 and Ser89 residues in response to the H100 treatment, indicating this two ARFs probably also can reduce the Cd accumulation and enhance Cd tolerance via transpiration rate or PCs synthesis in rice shoot.

In addition to ABA signaling, components of both CDPK and MAPK signaling were also phosphorylated by Cd stress. Of the seven rice CDPKs, the phosphorylation level of six was raised as a response to the H100 treatment (Tables S4 and S5). Two of these were CPK 13 (calcium-dependent protein kinase 13, Os07g0568600) and CDKC2 (cyclin-dependent kinase C 2, Os01g0958000). The former protein was observed to accumulate in both the rice leaf sheath and callus, and responds to low temperature and exogenous gibberellin by becoming increasingly phosphorylated [44]. The absence of CDKC2 enhances the plant’s tolerance to moisture stress through the re-programming of the transcription of genes encoding certain cell cycle-associated proteins; It also downgrades the phosphorylation status of RNA polymerase II, which impacts upon the development of stomata [45]. Here, the enhanced phosphorylation level of CDKC2 and CPK13 in response to the H100 treatment implied that both of these kinases participated in Cd detoxification. Stress-responsive MAP kinases are thought to be involved in the response to Cd [46]. The signaling protein DSM1/Os02g0743500 has been suggested to act as a MAP kinase during the response to moisture stress, perhaps by its regulation of ROS scavenging [35]. Here, the phosphorylation level of Q6Z2V3/Os02g0743500 was markedly enhanced in H100-treated seedlings, suggesting that it can confer Cd tolerance by regulation on stomata or scavenging ROS. In addition to the kinases and phosphatases involved in ABA, CaMK and MAPK signaling, in all over 50 differentially phosphorylated kinases or phosphatases were identified here: these included the RLK transmembrane protein kinase CrRLK1L4/Os01g0155500 and the leucine-rich repeat (LRR) protein kinase Q7XAK8/Os07g0106100. The overall conclusion was that multiple signaling pathways are likely involved in the response and tolerance to Cd stress.

### 3.2. Phosphorylated Transcription Factors Related to Stress Response and Defense

In response to external stress, the phosphorylation status of many transcription factors is altered through the action of protein kinases or phosphatases [47]. In rice challenged by drought stress, multiple serine-proline dipeptides in the transcription factor WRKY30 are phosphorylated [48]. It was revealed that majority of WRKYs (14 of 20 genes) were induced in *Populus* exposed to Cd stress [49]. In maize, WRKY4 was reported to enhance tolerance to Cd stress by increase in expression and activity of superoxide dismutase (SOD) and ascorbate peroxidase (APX) [50]. Here, both WRKY72/Os11g0490900 and WRKY1/Os01g0246700 responded to the H100 treatment became more strongly phosphorylated at, respectively, the residues Ser88 and Ser242/244 (Tables S4 and S5), suggesting that they both were probably involved in the plant’s defense against Cd stress by scavenging ROS. This was agreement with the literature revealing WRKY7 improved Cd tolerance through increase antioxidant enzymes SOD and peroxidase (POD) in *Arabidopsis* [51]. Similarly, the phosphorylation of WRKY72 has been associated with heightened salinity stress tolerance in rice [52]. Another class of transcription factor, the so-called Zn finger CCCH domain-containing proteins, has been implicated in cellular development and the abiotic stress response [53,54]. According Zhang et al. [22], changes in the phosphorylation level of two such transcription factors are a part of the response of wheat plants to moisture stress. In seedlings exposed to the H100 treatment, the Zn finger CCCH domain-containing C3H12/Os01g0917400, C3H20/Os03g0112700 and C3H45/Os06g0677700 proteins became more strongly phosphorylated. Other transcription factors, namely MYBc/Os09g0299200, bHLH113/Os10g0556200, MYC2/Os10g0575000, DDT/Os05g0562400 and the bZIPs Os01g0174000 and Os03g0239400 (Tables S4 and S5) were similarly more intensely phosphorylated in seedlings exposed to the H100 treatment, suggesting their involvement in the defense to Cd stress.

### 3.3. Phosphoproteins Classified as Stress-Related Proteins

Numerous phosphoproteins related to the general stress response have been shown to be induced in plants (particularly, cereals) exposed to abiotic stress [55,56]. Here, members of this large class of proteins, including heat shock proteins, chaperonins, E3 ubiquitin-protein ligase, oxidoreductase, peroxidases and Cd tolerance factor (Tables S4 and S5), were among the proteins which responded to the Cd stress by increasing their level of phosphorylation. SNAP32 (synaptosomal-associated protein 32)/Os02g0437200, a protein accumulated in response to various biotic and abiotic stresses [57], was more strongly phosphorylated in seedlings exposed to the H100 treatment than in those not challenged by Cd stress. Another example was represented by A0A0P0WJ59/Os05g0196100, a rice homolog of the multidrug tolerance associated protein 14 (MRP14): the Cd stress raised its phosphorylation level at the two residues Ser888 and Ser893, Which was supported by that MRPs (multidrug resistance-associated proteins) are thought to contribute to cellular detoxification, by transporting toxic compounds (such as Cd-phytochelatin complexes) from the cytosol into the vacuole [58]. Majority of proteins were damaged in plants under Cd stress. The chaperonins inhibit protein aggregation and refolding, while also activating other proteins required for protein folding and proteostasis [59]. Of the 19 chaperonins identified here, 15 responded to the H100 treatment by increasing their level of phosphorylation (Tables S4 and S5): these included HSP (heat shock protein) 70 and a 14-3-3-like protein GF14-E (G-box factor 14-3-3 homologs). The same phosphorylation response of HSP70 has been previously noted in rice plants exposed to moisture stress [33], while the up-regulation of the 14-3-3 like GF14-B phosphoprotein is thought to interact with the ubiquitin-dependent pathway in the context of protein degradation in salinity-challenged plants [60]. The ubiquitin protein degradation system relies on E3 proteins, many of which have been implicated in the response to abiotic stress [61]. Here, six such proteins, including Hrd1 (C3HC4-type ring finger domain containing protein 1)/Os06g0301000, responded to the H100 treatment by a rise in their phosphorylation level, as did two E3 proteins in response to moisture stress [33].

### 3.4. ROS-Related Phosphoproteins

Despite the fact that Cd is not directly involved in cellular redox reactions [62], Cd stress is known to enhance the production of ROS. Plants have evolved a suite of measures to limit the cytotoxicity of these oxidative compounds [63]. Here, ten distinct phosphoproteins were found to be associated with ROS scavenging, of which seven were identified in seedlings exposed to the H100, but not to either the M10 or NC treatments; these were a peroxidase (Os02g0192700), two NADH dehydrogenases (Os09g0500200 and Os05g0481600), two oxidoreductases (Os03g0862100 and Os08g0476300) and two thioredoxin-like proteins (Os01g0184800 and Os03g0767500) (Tables S4 and S5). Many gene products are involved in cellular homeostasis, which is a prerequisite for maintaining the morphological and physiological viability of an organism. One such protein is LHCB (light-harvesting chlorophyll a-b binding protein/Os07g0558400), which responded to the H100 treatment by increasing its level of phosphorylation at the residues Thr111 and Thr115 (Tables S4 and S5); this protein regulates the redox state of the plastoquinone mediating electron transfer between photosystems I and II [64]. A second electron transfer-related protein, which became more phosphorylated in response to the H100 treatment, was LFNR2 (leaf-type ferredoxin-NADP^+^ oxidoreductase 2)/Os02g0103800, a leaf-type ferredoxin NADP (nicotinamide adenine dinucleotide phosphate) reductase, which generates the reducing power required for numerous other reactions [65].

### 3.5. Phosphoproteins Involved in Water and Ion Transport

It has been shown that Cd stress can disturb the uptake, transport and use of water and several elements (Ca, Mg, P and K), resulting in osmotic pressure and ion unbalance [66]. Plants gradually adjust a series of transporters on the plasma membrane to defense Cd^2+^ toxicity. Aquaporins (AQPs) are plasma membrane intrinsic proteins (PIPs) that rapidly transport water induced by osmotic pressure [67]. In our study, three AQPs (Tables S4 and S5) were identified significantly changed in phosphorylation status in response to Cd stress, including two down-regulated DPs (PIP2-1/Os07g0448800, PIP2-6/Os04g0233400) in H100 treatment and one up-regulated DP (PIP2-7/Os09g0541000) in M10 treatment, indicating phosphorylation of AQPs may play an important function in this process.

Besides AQPs, another kind of transporter that is also associated with Cd^2+^ detoxication, ATP binding cassette (ABC)-type transporters is also involved in Cd^2+^ detoxication through transporting PCs-Cd into vacuole [68]. In the present study, two out of three ABC transporters (Tables S4 and S5), such as Q9ARU4/Os01g0121700 and ABC-2/Os03g06139, were identified up-phosphorylated in H100 treatment, indicating phosphorylation of ABC transporter probably enhanced its activity to transport Cd^2+^.

## 4. Materials and Methods

### 4.1. Rice Materials and Plant Growth Conditions

The cultivar used in these experiments was ‘Zhong Jiazao-17’ (Hangzhou, Zhejiang Province, China), a fast maturing *indica* type known to be a low accumulator of Cd in the grain. The grain was surface-sterilized by immersion in 5% sodium hypochlorite for 5 min, rinsed three times in sterile distilled water, imbibed for 24 h, then held at 30 °C for a further 24 h. Germinating seedlings were removed to a hydroponics solution consisting of Hoagland’s solution (pH 5.5), following [69]. Once the first leaf had fully expanded, seedlings were subjected to CdCl_2_·2.5H_2_O in different concentrations, including 0 μΜ (the control, NC), 10 μΜ (moderate stress, M10) and 100 μΜ (high stress, H100) for 12 days, when there were significantly different in growth and physiological traits among seedlings under this three Cd stress. Each treatment was represented by three replicates, each comprising a set of 32 uniformly sized seedlings. The seedlings were maintained in culture for 12 days under a relative humidity of 80%, with a day/night temperature regime of 30/28 °C.

### 4.2. Plant Growth and Chlorophyll Content Analysis

At the end of the Cd^2+^ treatment, the chlorophyll content of the youngest expanded leaf was measured using a SPAD (soil and plant analyzer development)-5 chlorophyll meter (Konika Minolta, Tokyo, Japan). The length of the shoot of each plant was recorded, and its dry weight measured after baking at 65 °C for 72 h.

### 4.3. Determination of Shoot Cd Contents

The Cd content of the shoots was determined as previously described [70]. Briefly, the shoots were rinsed in distilled water, dried by baking at 105 °C for 48 h and ground to a powder. About 100 mg the powder was digested in 5 mL 65% HNO_3_ at 60 °C for 48 h, and the resulting solution diluted by adding 20 volumes of Milli-Q water. The Cd concentration of the samples was derived by inductively coupled plasma-optical emission spectrometry, using an Optima 5300 V device (PerkinElmer, Inc., Waltham, MA, USA). Shoot Cd contents were expressed in the form mg per kg dry weight.

### 4.4. RNA Extraction and qRT-PCR Analysis

RNA was extracted from rice seedlings using the TRIzol reagent (Invitrogen, Carlsbad, CA, USA), and was treated with DNase I (Promega, Madison, WI, USA) to remove any contaminating genomic DNA. A 2 μL aliquot was reverse transcribed to cDNA using a PrimeScript^®^ RT reagent kit (Takara, Tokyo, Japan). Primer pairs for the subsequent qRT-PCR analyses (Table S7) were designed using Primer 5.0 software (PREMIER Biosoft International, Palo Alto, CA, USA) and checked specificity by blasting primer sequences in the NCBI database (Available online: www.ncbi.nlm.nih.gov/tools/primerblast/). The *Ubiquitin* gene (LOC4332169) was chosen as the reference. Each 20 μL qRT-PCR comprised 2 μL cDNA, 0.5 μL of each gene-specific primer, 9 μL 2.5× Real Master Mix/20× SYBR solutions and 8 μL ddH_2_O. The reactions were denatured at 95 °C for 3 min, and then cycled 40 times through 95 °C/20 s, 55 °C/10 s and 72 °C/20 s. At the end of the cycling process, a melting curve (65 °C to 95 °C) analysis was applied to check amplification specificity. The reactions were performed using a LightCycler 480 Real-time PCR Detection System (Roche, Basel, Switzerland).

### 4.5. Sample Preparation and iTRAQ Labeling

The root and the shoot of each seedling were separately snap-frozen in liquid nitrogen and stored at −80 °C. Protein was extracted from the shoot using the procedure given by Wang et al. [71] with the following minor modifications. In detail, approximately 500 mg of fresh leaves from each biological replicate were ground into a fine power in liquid nitrogen. Subsequently, the ground power was suspended in extraction buffer, and then added Postop Phosphatase Inhibitor Cocktail (one lablet/10 mL; Roche, Basel, Switzerland) and 1 mM phenylmethanesulfonyl fluoride (PMSF) to inhibit phosphatase activity and protease. The mixture was shaken vigorously for 30 s. After samples were centrifugated at 20,000× *g* and 4 °C for 30 min, protein supernatants were precipitated with four fold volumes of cold methanol plus 100 mM ammonium acetate. After centrifugation at 20,000× *g* and 4 °C for 20 min, the pellets were rinsed twice with cold acetone and then centrifugated so as to get protein mixtures. After freeze-drying was complete, the pellets were added to 300 μt of solubilization buffer at room temperature for 2 h. Finally, the concentration of protein samples were determined with a 2-D Quant Kit (Amersham Bioscience, Piscataway, NJ, USA), and final protein solution was stored at −80 °C until use. The protein concentrations of the sample set were equalized, and an aliquot of ca. 100 μg per sample was labeled using an iTRAQ device (Applied Biosystems, Foster City, CA, USA), applying the standard protocol provided with a 4-plex kit (126 and 127N/C, 128N/C and 129N, 129C and 130N/C for the control, 10 μΜ and 100 μΜ separately).

### 4.6. Enrichment for Phosphorylated Peptides

Labeled peptides were mixed, concentrated by vacuum evaporation and resuspended in 500 μL of loading buffer (65% acetonitrile (CAN), 2% *w*/*v* glutamic acid and 2% trifluoro acetic acid (TFA)). TiO_2_ beads were added; the mixture was agitated for 40 min, and then centrifuged (5000× *g*, 1 min). The procedure was repeated with the resulting supernatant and the two sets of beads were combined and rinsed three times in 50 μL of 30% CAN, 3% TFA, then a further three times in 50 μL of 80% CAN, 0.3% TFA. Phosphopeptides were eluted from the beads by adding 50 μL of 40% CAN, 15% NH_4_OH. The eluate was lyophilized.

### 4.7. Liquid Chromatography Tandem-Mass Spectrometry (LC-MS/MS) Analysis

For the purposes of LC-MS/MS, 5 μL of phosphopeptide solution was mixed with 15 μL of 0.1% (*v*/*v*) TFA, and a 10 μL aliquot of this mixture was loaded into a Q Exactive mass spectrometer (Thermo Finnigan, Somerset, NJ, USA) coupled to an Easy-nLC 1200 liquid chromatograph (Thermo Fisher Scientific, Waltham, MA, USA). The C18-reversed phase column dimensions were: length 25 cm, inner diameter 75 μM, RP-C18 3 μΜ. The elution buffer was a mixture of 0.1% formic acid in 2% *v/v* acetonitrile (A) and 0.1% formic acid in 80% *v/v* acetonitrile (B) at a flow rate of 300 nL/min over 155 min. Over the period 0–101 min, the concentration of B rose linearly from 0% to 19% B, from 101–136 min; it was increased from 19% to 29%, from 136–142 min from 29% to 38% and from 142–155 min from 38% to 100%. For the mass spectrometry, the positive ion mode was adopted, with peptide recognition mode enabled. The data were acquired using a data-dependent top 20 method, achieved by choosing the most abundant precursor ions from the survey scan (350–1300 *m*/*z*) for HCD (higher-energy C-trap dissociation) fragmentation. The range of charge was from +2 to +6. Target values were determined by predictive automatic gain control. The dynamic exclusion duration was 18 s. Survey scans were acquired at a resolution of 70,000 at 200 *m*/*z* and the resolution set for the HCD spectra was 35,000 at 200 *m/z*. The normalized collision energy was 30 eV and the underfill ratio was defined as 0.1%. Three technical replicates were performed for each sample.

### 4.8. Phosphopeptide and Phosphosite Identification

The Uniprot_*Oryza sativa* database (Avalable online: http://www.uniprot.org/proteomes/) and a decoy database was searched for matches to the acquired MS/MS spectra using Mascot v2.2 software embedded in Proteome Discoverer 2.1 (Thermo Fisher Scientific, Waltham, MA, USA). The parameters applied for protein identification were: MS/MS tolerance = 0.02 Da; peptide mass tolerance = 10 ppm; missed cleavage = 2; enzyme = trypsin; fixed modification: iTRAQ4plex (N-term); iTRAQ4plex (K); carbamidomethyl (C); phosphorylation (S/T/Y) and variable modification: oxidation (M). The false discovery rate threshold for both peptides and proteins was set as 1.0%. The probability that a given phosphorylation site was truly phosphorylated was used to evaluate the PhosphoRS site probability: probabilities >75% were taken as evidence of true phosphorylation. Phosphopeptide ratios were normalized against the average value of all identified peptides. The quantification of phosphopeptides was represented by the mean value of three biological replicates. Statistical significance between means was assigned using the Students’ *t*-test. A Benjamin-Hochberg false discovery rate was applied in multiple comparisons. Significant changes in a phosphopeptide’s abundance were inferred where its abundance ratio was >1.2 or <0.83 and its *p*-value derived from the Students’ *t*-test was <0.05.

### 4.9. Bioinformatics

GO annotation and enrichment were conducted using the AgriGO tool [72]. “Eukaryotes” database of CELLO (Available online: http://cello.life.nctu.edu.tw/) was used to identify the subcellular localization of proteins. Phosphorylation site motifs and the specificity of these motifs were predicted using Motif-X online software (Available online: motif-x.med.harvard.edu/motif-x.html). KOG (cluster of orthologous groups of proteins for eucaryon) numbers were obtained from a search of the EggNog database (Available online: eggnog.embl.de/version_4.0.beta/). STRING v10.0 software (Available online: http://string-db.org/) was deployed to identify potential protein-protein interactions between sets of phosphorylated proteins, applying a confidence score of 0.75. The resulting inferred networks were visualized using Cytoscape v3.0 software (Available online: http://www.cytoscape.org).

## 5. Conclusions

Overall, the experiment revealed a set of 1244 proteins (harboring 2454 phosphosites) as responding to Cd stress. Of the 482 that were differentially phosphorylated, 392 were more strongly phosphorylated by exposure to Cd^2+^, 98% of which were identified in the H100 treatment. A functional analysis suggested that the proteins which responded to the stress by becoming more highly phosphorylated included several involved in ABA signaling (PP2C66, PP2C30, Q6ZI44, Q0J0U2, Q18PR9, Q94DL7 and Q2R4Z4), in CDPK signaling (CPK13 and Q5JK68) and in MAPK signaling (DSM1); another notable group comprised the transcription factors WRKY72, WRKY1, C3H12, C3H20 and C3H45. The gene products associated with abiotic stress tolerance included SNAP32, MRP14, HSP70, the 14-3-3-like protein GF14-E and various E3 proteins. Other gene products liable to become phosphorylated were Hrd1 (Os06g0301000), a peroxidase (Os02g0192700), two NADH dehydrogenases (Os09g0500200 and Os05g0481600), two oxidoreductases (Os03g0862100 and Os08g0476300), two thioredoxin-like proteins (Os01g0184800 and Os03g0767500), LHCB (light-harvesting chlorophyll a/b-binding protein) and LFNR2 (Leaf-type ferredoxin-NADP+ oxidoreductase 2). All of these products likely are involved in the rice seedling response to Cd stress. This study represents a first attempt to use quantitative phosphoproteome analysis to reveal the molecular basis of the response to Cd^2+^ exposure, and the data which have emerged will contribute to a better understanding of the contribution of phosphorylation to Cd stress tolerance and detoxification in rice.

## Supplementary Materials

Supplementary materials can be found at www.mdpi.com/1422-0067/18/10/2055/s1.
