# Supplementary material for: The Phosphoproteomic Response of Rice Seedlings to Cadmium Stress
Source: Int J Mol Sci. 2017 Sep 27;18(10):2055. doi: 10.3390/ijms18102055 (PMC5666737; doi:10.3390/ijms18102055)
Supplement: Supplementary file 1 [file ijms-18-02055-s001.zip › Additional Figure S1, S2.pdf]

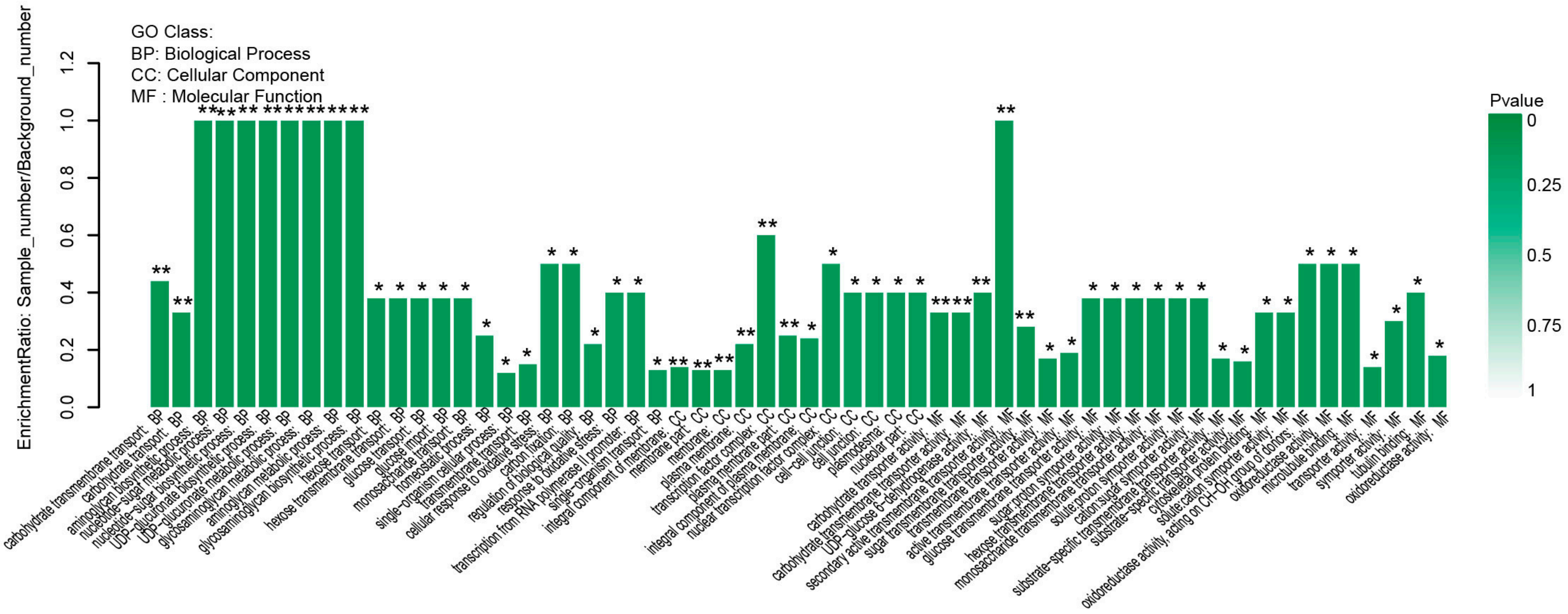

Additional Figure S1: Gene ontology enrich analysis of the differentially phosphorylated proteins in shoot in response to M10 treatment. *, **: means differ significantly from one another at, respectively P <0.05 and P <0.01.

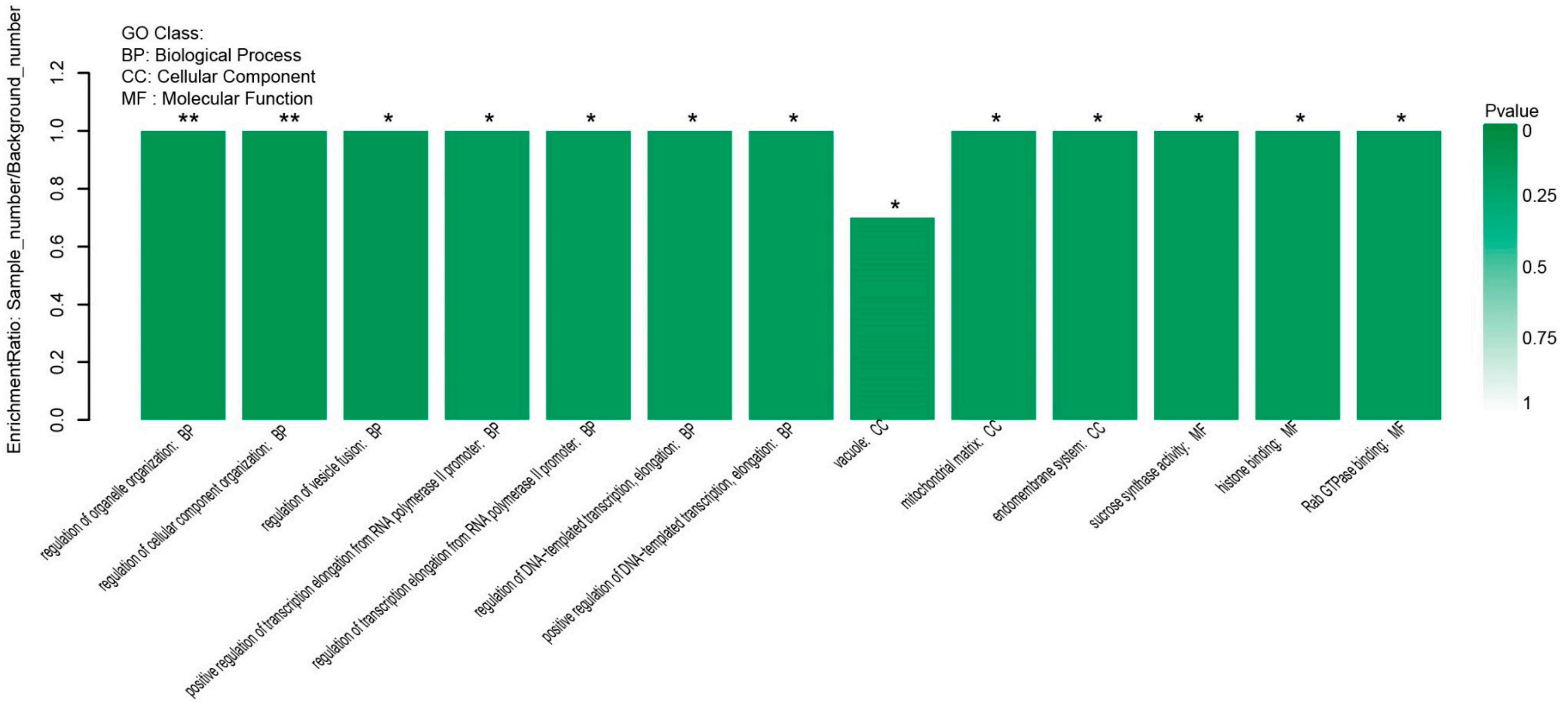

Additional Figure S2: Gene ontology enrich analysis of the differentially phosphorylated proteins in shoot in response to H100 treatment. *, **: means differ significantly from one another at, respectively $P < 0.05$ and $P < 0.01$.
